# Patient Population and Test Utilization for Thyroid Function in Local Clinics and Hospitals in Korea

**DOI:** 10.3390/diagnostics12071638

**Published:** 2022-07-05

**Authors:** Rihwa Choi, Sang Gon Lee, Eun Hee Lee

**Affiliations:** 1 Department of Laboratory Medicine, Green Cross Laboratories, Yongin 16924, Korea; pirate0720@naver.com; 2 Department of Laboratory Medicine and Genetics, Samsung Medical Center, Sungkyunkwan University School of Medicine, Seoul 06351, Korea; 3 Green Cross Laboratories, Yongin 16924, Korea

**Keywords:** thyroid function test, test utilization, local clinics, Korea

## Abstract

We evaluated the utilization and characteristics of thyroid function test (TFT) results, including serum thyroid stimulating hormone (TSH), free thyroxine (free T4), and total triiodothyronine (total T3) in Korean adults who visited local clinics and hospitals between 2018 and 2020. We obtained TFT results for 69,575 specimens from 47,685 adult Korean patients (4878 men and 42,807 women) with a mean age of 42.7 (standard deviation of 13.2) years. Among them, 23,581 specimens were tested for TSH only, 38,447 specimens were tested for TSH and free T4 (including 17,978 specimens without total T3), and 20,469 specimens were tested for all three, i.e., TSH, free T4, and total T3. The proportion of euthyroid was 80.0% among all 69,575 specimens, 71.2% among specimens with TSH and free T4, and 64.2% among specimens with all three TFTs. TFT patterns that were difficult to interpret and needed more clinical information were observed for about 6.9% of the 20,469 specimens with all three TFTs. Among the 20,469 specimens with all three TFTs, no specimen had increases in all three. Information on the prevalence of specimen results of TFTs would be helpful to expand our knowledge of patient population characteristics and to improve test utilization.

## 1. Introduction

Thyroid function tests (TFTs) are one of the most frequently utilized tests by physicians in clinical laboratories [1]. The physiology of thyroid hormones is based on the hypothalamic–pituitary–thyroid axis, which includes the control of thyroid function subject to negative feedback [2]. Thyroid function tests include the measurable thyroid stimulating hormone (TSH), total thyroxine (total T4), free thyroxine (Free T4), total triiodothyronine (total T3), free triiodothyronine (free T3), reverse T3, thyroglobulin, thyroxine-binding globulin, and various types of antibody tests to define characteristics of thyroid disorders [1,3]. Among these TFTs, several clinical practice guidelines on thyroid diseases are available, with TSH recommended as the initial screening test for thyroid function and the additional use of free T4 and/or total T3 (in Korea and United States) [4,5,6,7] or free T3 tests (European countries) for further assessment [1,8,9]. In Korea, both serum TSH and free T4 tests are recommended by the Korean Thyroid Association at the time of the initial evaluation when hyperthyroidism is strongly suspected, and total T3 measurement is helpful for the diagnosis of T3 toxicosis [4]. Furthermore, the 2011 guidelines from the American Thyroid Association and American Association of Clinical Endocrinologists also suggested that diagnostic accuracy improves when both serum TSH and free T4 are assessed at the time of the initial evaluation when hyperthyroidism is strongly suspected [10]. The 2016 update indicated that the diagnostic accuracy improves when serum TSH, free T4, and total T3 are assessed at the initial evaluation [7]. The European Thyroid Association reported similar increased diagnostic accuracy for both serum TSH and free T4 at the time of initial evaluation; however, the importance of serum free T3 is mentioned for milder hyperthyroidism, in which serum total T4 and free T4 levels can be normal, and only serum free T3 may be elevated, with an undetectable serum TSH in milder hyperthyroidism [11].

Combinations of different TFT results can be interpreted together with patient clinical information [1,2,3]. In some cases, physicians can have difficulty in interpreting laboratory results with different combinations of increased and/or decreased results of each TFT. Confusing TFT results can be caused by characteristics of physiological changes for each hormone, samples of patients with and without thyroid illness, and by interference caused by characteristics of analytical methods [1,2]. It is important to understand the characteristics of a patient population, including the prevalence of diseases, to understand and evaluate the clinical performance of laboratory tests and to improve the quality of clinical testing [1,6].

Most previous studies regarding TFTs in Korea have been performed using populations visiting university hospitals. Few studies have involved the comprehensive analysis of laboratory results for TFT utilization in a Korean population visiting local clinics and hospitals. Green Cross Laboratories is one of the largest referral clinical laboratories in Korea, providing a TFT testing service to local clinics and hospitals throughout Korea.

Therefore, the aim of this study is to provide information on the utilization of TFTs in local clinics and hospitals in Korea and the prevalence of combinations of TFTs to help physicians and clinical laboratory professionals understand patient population characteristics in Korea.

## 2. Materials and Methods

We obtained TFT results from adult Korean patients (>20.0 years) who visited local clinics and hospitals and underwent serum TFTs (TSH, free T4, and total T3) through the laboratory information system of Green Cross Laboratories between January 2018 and December 2020. As Green Cross Laboratories is a referral laboratory with limited access to clinical information, possible interpretation of TFTs was based on the increase or decrease of each hormone. All data were anonymized before analysis. Data missing age or sex were excluded. This study was conducted according to the guidelines expressed in the Declaration of Helsinki, and all procedures involving human subjects were approved by the Institutional Review Board of Green Cross Laboratories (GCL-2022-1017-01), which waived the need for informed consent for the retrospective data collection and review.

Serum TFTs were performed using an automated electrochemiluminescence immunoassay on Cobas e801 analyzers of the Cobas 8000 system (Roche, Mannheim, Germany). Serum TSH, free T4, and total T3 analyses were performed using Elecsys TSH (measuring range of 0.005–100 μIU/mL), Elecsys FT4 II (measuring range of 0.04–7.76 ng/dL), and Elecsys T3 (0.2–6.5 ng/mL) assay kits according to the manufacturer’s instructions. Reference intervals for TFTs were applied as follows: 0.27–4.20 μIU/mL for TSH, 0.93–1.70 ng/dL for free T4, and 0.8–2.0 ng/mL for total T3. For test results of each hormone outside the lower or upper reference limits, “decreased (↓)” or “increased (↑)” was labeled, respectively. Possible interpretation of the TFT results pattern was applied according to previous studies [12,13]. When results of the TSH, free T4, and/or total T3 pattern were not matched to usual patterns, the specimen was categorized as ‘more clinical information needed’ (on treatment for thyroidal illness, nonthyroidal illness, subacute thyroiditis, T4 protein-binding abnormalities, medication, interference, etc.) [1,2].

Categorical variables (increase or decrease of each hormone and possible interpretation) were presented as frequencies and percentages. The Chi-square test was used for comparison of categorical variables (age group and TFT pattern). Mann–Whitney U tests were used for the comparison of nonparametric quantitative variables (age, number of follow-up TFTs, and TFT levels) by sex and age groups. Here, *p*-values < 0.05 were considered statistically significant. Statistical analysis was performed using MedCalc Statistical Software version 20.110 (MedCalc Software bv, Ostend, Belgium; https://www.medcalc.org; accessed on 29 June 2022).

## 3. Results

### 3.1. Baseline Characteristics of the Study Subjects

During the three-year study period, we obtained TFT results for 69,575 specimens from 47,685 adult Korean patients (4878 mean and 42,807 women) with a mean age of 42.7 (standard deviation of 13.2) years (Table 1). Among them, 23,581 (33.9%) specimens were tested for TSH only, 38,447 (55.3%) specimens were tested for TSH and free T4 (including 17,978 specimens without total T3), and 20,469 (29.4%) specimens were tested for all three TSH, free T4, and total T3. Among all patients, 37,449 (53.8%) subjects had only one measure of TFT without follow-up. Women aged 30 to 39 years were the most prevalent (45.5%) in this study. Among men, subjects aged 50–59 years were the most prevalent age group (29.7%). Median age, TSH, and free T4 levels at baseline were significantly different by sex (*p* < 0.001, 0.001, <0.001, respectively), while number of follow-up TFTs and total T3 levels were not significantly different (*p* > 0.05).

### 3.2. Thyroid Function Test Results and Possible Interpretation

**Figure 1:**
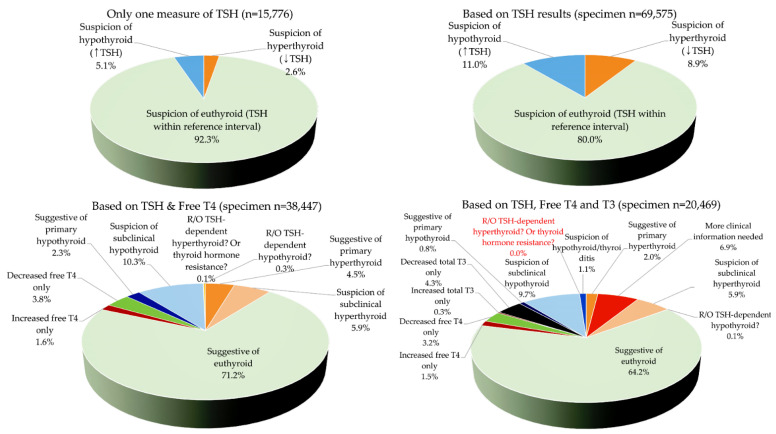
Prevalence of specimen thyroid function test (TFT) results. Interpretation of results among 15,776 specimens from 15,776 patients who had only one TSH measurement (**left upper**), interpretation using TSH results only among all 69,575 specimens (**right upper**), interpretation using TSH and free T4 among 38,447 specimens tested for both TSH and free T4 (**left lower**), and interpretation using TSH, free T4, and total T3 among 20,469 specimens tested for all three TFTs (**right lower**). Specimens needing more clinical information included possible cases for patients being treated for thyroidal illness, nonthyroidal illness, subacute thyroiditis, thyroxine protein-binding abnormalities, possible interference, etc. Schemes follow the same formatting. (↑) represents ‘increased’ and (↓) represents ‘decreased.’

The TFT results and possible interpretations are summarized in Table 2 and Figure 1. From all 47,685 subjects, 15,776 (33.1%) had only one measure of TSH test; among these, 2.6% (414/15,776) had decreased TSH (suspicion of hyperthyroid) and 5.1% (798/15,776) had increased TSH (suspicion of hypothyroid, Figure 1). For all 69,575 specimens, decreased TSH (suspicion of hyperthyroid) and increased TSH (suspicion of hypothyroid) were found in 8.9% and 11.0%, respectively (euthyroid with normal TSH, 80.0%). Among 38,447 specimens with TSH and free T4 results, the proportion of euthyroid was 71.2%; for 20,469 specimens with all of three TFTs, the proportion of euthyroid was 64.2%. TFT patterns that were difficult to interpret and required more clinical information, including whether specimens were from patients being treated for thyroidal illness, nonthyroidal illness, subacute thyroiditis, T4 protein-binding abnormalities, medication use, or possible interference, were observed for about 6.9% of 20,469 specimens with all three TFTs. No specimens with all three TFTs showed an increase in all three.

### 3.3. Thyroid Function Test Results and Possible Interpretation by Sex and Age

**Figure 2:**
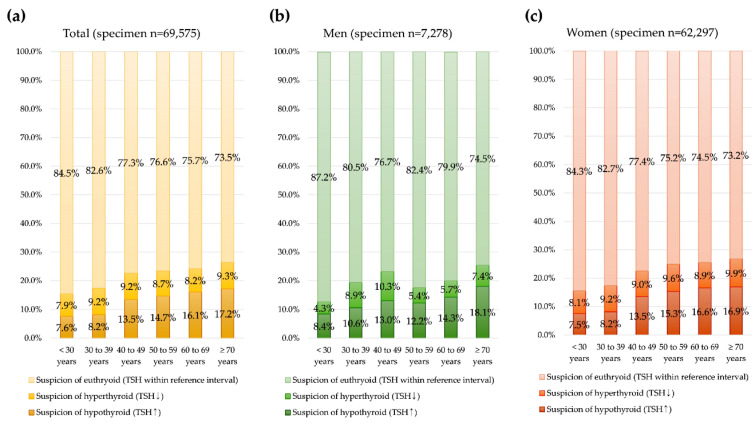
Specimen thyroid function test (TFT) results by age group. (**a**) For 69,575 overall specimens, (**b**) men, and (**c**) women.

**Figure 3:**
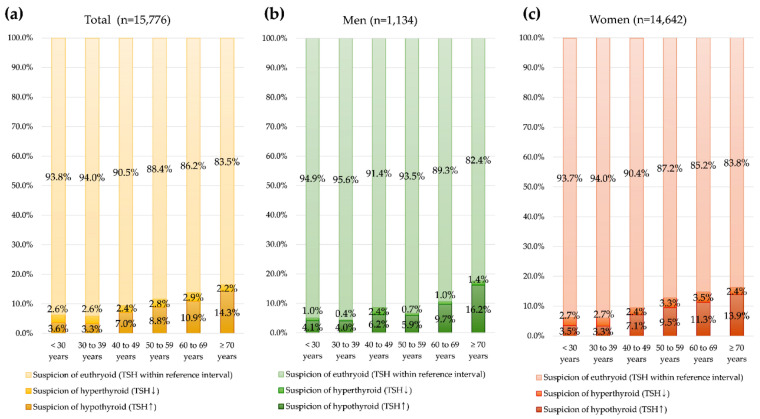
Specimen thyroid function test (TFT) results among 15,776 specimens with only one measure of TSH test by age group. (**a**) For total subjects, (**b**) men, and (**c**) women.

The TFT results and possible interpretations for 69,575 total specimens and by sex are summarized in Figure 2. The specimen prevalence of increased TSH (suspicion of hypo-thyroid) and decreased TSH (suspicion of hyperthyroid) was significantly different by sex and age group (*p* < 0.001). The increased TSH (suspicion of hypothyroid) specimen prevalence was highest in patients age ≥ 70 years. Among 15,776 specimens with only one TSH test, the same pattern with increased prevalence of high TSH (suspicion of hypothyroid) by age was observed (Figure 3).

**Figure 4:**
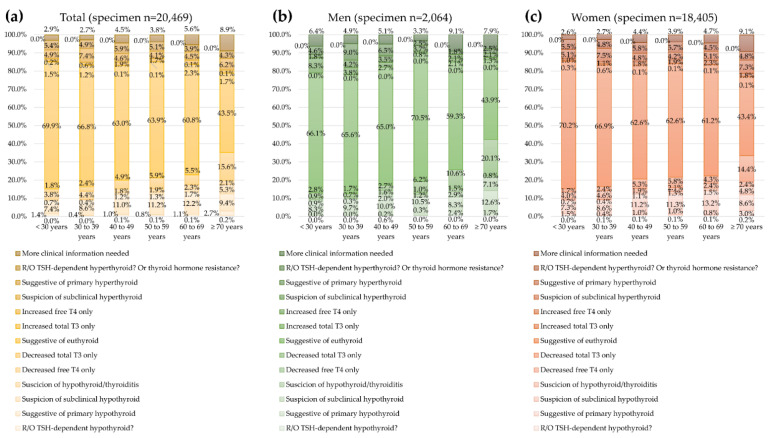
Prevalence of specimen thyroid function test (TFT) results among 20,469 specimens with all three TFTs by age group. (**a**) For total specimens, (**b**) men, and (**c**) women.

For 20,469 specimens with all three TFTs, the proportion of euthyroid decreased with an increasing age (Figure 4). The proportion of euthyroid was the highest in men aged 50–59 years, which was the predominant age group among men. The prevalence of specimens with “more clinical information needed” was higher in men (5.6%) than in women (3.6%).

## 4. Discussion

In this study, we have evaluated the test utilization and prevalence of serum TFTs with possible interpretations according to combinations of increased or decreased hormones in adult Korean patients who visited local clinics and hospitals. In this study, more women were tested for TFTs, which is consistent with previous studies that found thyroid diseases to be more prevalent in women [1,14].

Laboratory tests and thyroid imaging tests, such as thyroid ultrasound or radionuclide scans, are integral in the diagnosis and management of various types of thyroid dis-eases [15]. Immunoassay techniques for TFTs are widely used in clinical laboratories for the measurement of thyroid function tests, with the aid of full automation, short turn-around time, and high specificity and sensitivity for routine clinical practice [16]. TFT results from immunoassays should be interpreted cautiously, because the results can be affected by interference, concurrent medications, pregnancy, non-thyroidal illness, and age [15]. Interpretation of TFT results is one of the areas of clinical chemistry that has a potential to improve patient outcomes and to reduce the risk of errors [17].

In this study, about one-third of all tested patients had only one measure of TSH without a follow-up measure during the three-year period, which is the population that might be screened for thyroid function. The proportion of this group on suspicion of euthyroid (TSH level within reference interval) was 92.8% in this population. Meanwhile, the proportion of euthyroid decreased with the number of TFT increases; 71.2% among specimens with TSH and free T4 tests and 64.2% with all three TFTs. Although clinical information was limited for this study, this finding suggests that patients with or suspected of thyroidal disease are monitored using additional thyroid hormones [1]. It has been reported that the prevalence of hypothyroidism including subclinical forms in adults is 5–15%, and hyperthyroidism is seen in 0.5–2% of the population.1,12 In this study, among 15,776 patients who had only one TSH test result, 5.1% (798/15,776) had increased TSH (suspicion of hypothyroid) and 2.6% (414/15,776) had decreased TSH (suspicion of hyperthyroid), results that are comparable with previous findings [1].

Additional thyroid hormone testing could provide additional clues for a clinical status of thyroid disease [1,7,9,13]. According to the clinical practice guidelines of the Korean Thyroid Association (KTA), measurement of both serum TSH and free T4 levels at the time of initial evaluation and total T3 measurement are helpful for diagnosis of T3-toxicosis when hyperthyroidism is strongly suspected [4]. In European countries, TSH, free T4, and free T3 tests are recommended when thyroid dysfunction is suspected [3,8,9,11]. In this study, additional free T4 tests provided additional information in about 22.2% of specimens tested for TSH and free T4, and additional total T3 tests gave additional information in about 7.7% of specimens tested for TSH, free T4, and total T3. Meanwhile, 6.9% of specimens tested for TSH, free T4, and total T3 showed confusing patterns that needed additional clinical information including whether patients were being treated for thyroidal illness, nonthyroidal illness, subacute thyroiditis, thyroxine protein-binding abnormalities, possible interference, etc. [1,2]. This group of patients may represent the cause of physician difficulties in interpreting laboratory results, leading to follow-up and/or further tests to determine the clinical significance of TFTs. In clinical laboratories, knowledge of the prevalence of unusual results is important for risk management and to improve clinical laboratory service [6]. Information on the prevalence of specimens with specific test results can be used to evaluate the performance of a clinical test in the laboratory [18,19]. In this study, none of the 20,469 specimens showed increases in all TSH, free T4, and total T3. As specimens with this pattern were extremely rare among samples from patients visiting local clinics and hospitals, repetitive, follow-up, and further tests likely will be required to rule out analytical errors, if this type of specimens is identified.

In Korea, the public database (Healthcare Bigdata Hub) for utilization of reimbursed clinical tests by the Health Insurance Review & Assessment Service (HIRA) only provides information of the numbers of thyroid hormone tests, which are regarded as one code for “thyroid hormone” (except TSH), and information of individual hormone tests could not be obtained through the database. A strength of this study is that the individual hormones free T4 and total T3 utilized with TSH were investigated through large numbers of data for Korean adults.

A limitation of this study was the lack of clinical information including detailed medical history, physical examination, other laboratory and radiological studies associated with thyroidal diseases and comorbidities affecting thyroid hormones. However, since symptoms of thyroid dysfunction are nonspecific, and the pattern of TFTs is used as a basis for suspicion of thyroid hormone status by clinical guidelines for thyroidal diseases, this study provides valuable information regarding the clinical situation and use of monitoring test. This approach can help clinical laboratories with limited clinical information to understand characteristics of their specimen population [1]. Future studies on the clinical impact of TFT utilization and prevalence of disease population are needed.

## 5. Conclusions

In conclusion, we have investigated the utilization and prevalence of TFT results for specimens requested from local clinics and hospitals in Korea. Women underwent TFTs more than men. About half of overall specimens tested free T4 and about half of those specimens were tested additionally for total T3. None of tested specimens showed increases in all three measures (TSH, free T4, and total T3), which may be useful information for clinical laboratories, as such patterns should be further investigated with detailed clinical information for analytical errors. Information on the prevalence of specimens with specific test results can be used to evaluate the performance of a clinical test in the laboratory. Evaluation of clinical laboratory specimen prevalence by specific characteristic can help to expand our knowledge of patient population characteristics. This can act as baseline information used to detect unusual findings in routine clinical practice and prevent errors in clinical laboratories.

## Figures and Tables

**Table 1:** Baseline characteristics of 47,685 study subjects.

Characteristics	Total Subjects (*n* = 47,685)	Men (*n* = 4878)	Women (*n* = 42,807)
Age, years (median, IQR)	37.8 (33.0 to 51.8)	52.7 (41.3 to 60.8)	36.9 (32.7 to 49.6)
Age group (*n*, %)			
<30 years	4755 (10.0%)	326 (6.7%)	4429 (10.3%)
30–39 years	22,483 (47.1%)	788 (16.2%)	21,695 (50.7%)
40–49 years	7268 (15.2%)	981 (20.1%)	6287 (14.7%)
50–59 years	7209 (15.1%)	1448 (29.7%)	5761 (13.5%)
60–69 years	3904 (8.2%)	875 (17.9%)	3029 (7.1%)
70 years	2066 (4.3%)	460 (9.4%)	1606 (3.8%)
Follow-up, number (median, IQR)	1 (1 to 1)	1 (1 to 1)	1 (1 to 1)
Baseline thyroid function test result			
TSH, μIU/mL (median, IQR)	1.78 (1.08 to 2.78)	1.79 (1.15 to 2.79)	1.77 (1.07 to 2.78)
Free T4, ng/dL (median, IQR)	1.25 (1.12 to 1.39)	1.30 (1.17 to 1.45)	1.24 (1.11 to 1.38)
Total T3, ng/mL (median, IQR)	1.00 (0.90 to 1.20)	1.10 (0.90 to 1.20)	1.00 (0.90 to 1.20)

Abbreviations: IQR, interquartile range; TSH, thyroid stimulating hormone; T4, thyroxine; T3, triiodothyronine.

**Table 2:** Prevalence of thyroid function test results.

TFT	TSH	Free T4	Total T3	Possible Interpretation	*n*	%
TSH only (*n* = 69,575)	↓	N/A	N/A	Suspicion of hyperthyroid	6207	8.9
	-	N/A	N/A	Suspicion of euthyroid	55,686	80.0
	↑	N/A	N/A	Suspicion of hypothyroid	7682	11.0
TSH & Free T4 (*n* = 38,447)	↓	↑	N/A	Suggestive of primary hyperthyroid	1745	4.5
	↓	-	N/A	Suspicion of subclinical hyperthyroid	2252	5.9
	-	-	N/A	Suggestive of euthyroid	27,387	71.2
	-	↑	N/A	Increased free T4 only	630	1.6
	-	↓	N/A	Decreased free T4 only	1443	3.8
	↑	↓	N/A	Suggestive of primary hypothyroid	870	2.3
	↑	-	N/A	Suspicion of subclinical hypothyroid	3979	10.3
	↑	↑	N/A	R/O TSH dependent hyperthyroid? Or thyroid hormone resistance?	38	0.1
	↓	↓	N/A	R/O TSH-dependent hypothyroid?	103	0.3
TSH, Free T4 & Total T3 (*n* = 20,469)	↓	↑	↑	Suggestive of primary hyperthyroid	400	2.0
	↓	↑	-	Suggestive of primary hyperthyroid	628	3.1
	↓	↑	↓	More clinical information needed ^a^	15	0.1
	↓	-	-	Suspicion of subclinical hyperthyroid	1202	5.9
	↓	-	↑	More clinical information needed ^a^	65	0.3
	↓	-	↓	More clinical information needed ^a^	74	0.4
	↓	↓	↓	R/O TSH-dependent hypothyroid?	14	0.1
	↓	↓	-	More clinical information needed ^a^	58	0.3
	↓	↓	↑	More clinical information needed ^a^	2	<0.1
	-	-	-	Suggestive of euthyroid	13,131	64.2
	-	↓	-	Increased free T4 only	314	1.5
	-	↑	-	Decreased free T4 only	660	3.2
	-	-	↑	Increased total T3 only	65	0.3
	-	-	↓	Decreased total T3 only	889	4.3
	-	↓	↓	More clinical information needed ^a^	111	0.5
	-	↑	↑	More clinical information needed ^a^	3	0.0
	-	↑	↓	More clinical information needed ^a^	47	0.2
	-	↓	↑	More clinical information needed ^a^	10	<0.1
	↑	↓	↓	Suggestive of primary hypothyroid	171	0.8
	↑	↓	-	More clinical information needed ^a^	364	1.8
	↑	↓	↑	More clinical information needed ^a^	3	<0.1
	↑	↑	↑	R/O TSH-dependent hyperthyroid? Or thyroid hormone resistance?	0	0.0
	↑	↑	-	More clinical information needed ^a^	15	0.1
	↑	↑	↓	More clinical information needed ^a^	6	<0.1
	↑	-	-	Suspicion of subclinical hypothyroid	1987	9.7
	↑	-	↑	More clinical information needed ^a^	7	<0.1
	↑	-	↓	Suspicion of hypothyroid/thyroiditis	228	1.1

Abbreviation: N/A, not available; TFT, thyroid function test; TSH, thyroid stimulating hormone; free T4, free thyroxine; T3, triiodothyronine. (↓) represents ‘decreased,’ (↑) represents ‘increased.’ And (-) represents ‘within reference interval’. ^a^ Patients on treatment for thyroidal illness, nonthyroidal illness, subacute thyroiditis, thyroxine protein-binding abnormalities, medication use, possible interference, etc.

## Data Availability

The datasets generated and analyzed during the current study are available from the corresponding authors on reasonable request.
